# Moderate level platelet count might be a good prognostic indicator for intra-abdominal infection in acute pancreatitis: A retrospective cohort study of 1,363 patients

**DOI:** 10.3389/fmed.2022.1077076

**Published:** 2023-01-09

**Authors:** Wenwu Sun, Jun Huang, Tongtian Ni, Yi Wen, Gui Menglu, Wang Yongguo, Zhao Yanbin, Huiqiu Sheng, Ying Chen, Li Ma, Bing Zhao, Enqiang Mao

**Affiliations:** ^1^Department of Emergency, Ruijin Hospital, School of Medicine, Shanghai Jiao Tong University, Shanghai, China; ^2^Shanghai Key Laboratory of Hypertension, Department of Hypertension, Ruijin Hospital, Shanghai Institute of Hypertension, Shanghai Jiao Tong University School of Medicine, Shanghai, China; ^3^Department of Anesthesiology and Intensive Care, Linze County People’s Hospital, Zhangye, Gansu, China; ^4^Department of Anesthesiology, Maternity and Children Hospital, Linxia, Gansu, China

**Keywords:** acute pancreatitis, platelet count, infection, mortality, retrospective study

## Abstract

**Background:**

Early recognition of the risk factors is important for acute pancreatitis management. The aim of this study is to investigate the relationship between platelet count and clinical outcomes in patients with acute pancreatitis.

**Methods:**

The data are collected from a university-affiliated hospital between January 2013 and December 2020. A generalized additive model and a two-piecewise linear regression model are used to estimate the association between platelet count and the risks of intra-abdominal infection, surgical intervention, in-hospital mortality, and length of hospital stay.

**Results:**

Among the 1,363 patients, 99 (7.3%) patients suffered intra-abdominal infection, 190 (13.9%) patients underwent surgical intervention, and 38 (2.8%) patients died in the hospital. The median length of hospital stay is 21 days. Generalized additive model and two-piecewise linear regression analysis show that the risk of intra-abdominal infection decreases as the platelet count increases to 160 × 10^9^/L (OR: 0.991, 95% CI: 0.984–0.998, *p* = 0.015) and then increases as the platelet count levels up (OR: 1.007, 95% CI: 1.004–1.010, *p* < 0.001). The trend is similar to the risk of surgical intervention and length of hospital stay. Even though there seems a declining trend in mortality, no significant association is found after adjustment for potential confounders. Further analysis shows that changes in platelet count within the first 3 days after admission have no obvious association with clinical outcomes.

**Conclusion:**

A platelet count of approximately 160 × 10^9^/L on admission is associated with the lowest risk of intra-abdominal infection, surgical intervention, and shortest hospital stay in patients with acute pancreatitis.

## Introduction

Acute pancreatitis (AP) is a common gastrointestinal disease with an annual incidence of 34 per 100,000 person-years (1, 2). Activation of the pancreatic enzymes within the injured pancreatic acini is the most widely accepted pathophysiological theory. After the onset of pancreatitis at the early stage, uncontrolled oxidative stress and large amounts of released inflammatory cytokines induce systemic inflammatory response syndrome (SIRS) and contribute to progressive organ dysfunction (3). Most patients present with a mild clinical course, but organ dysfunction progresses in approximately 20% of the patients. The overall mortality rate remained as high as 10–20% in these patients (4).

Microcirculatory dysfunction plays an important role in the pathogenesis of AP. Uncontrolled oxidative stress and released inflammatory cytokines lead to endothelial injury and the activation of coagulation (5). Abnormal coagulation is manifested by vascular leakage, scattered intravascular thrombosis, and disseminated intravascular coagulation and is associated with increased complications and mortality in AP (6). In general, the platelet is defined as a pivotal factor in homeostasis and thrombosis. It also has been recognized as a member of the immune system and plays a role in inflammation response. Abnormal platelet features are common clinical manifestations associated with poor outcomes in patients who are critically ill (7, 8). However, the role of platelet count in AP pathophysiology has not been elucidated. Here, a retrospective hospital-based cohort study is conducted in patients with AP to evaluate the association between platelet count and clinical outcomes, especially the risk of intra-abdominal infection.

## Methods and analysis

### Participants

This retrospective study was approved by the institutional ethics board of Ruijin Hospital, Shanghai Jiao Tong University School of Medicine, and the informed consent was waived. Data analysis was performed on the 1964 Helsinki Declaration and its later amendments.

Consecutive adult patients (age ≥ 18 years) discharged with a diagnosis of acute pancreatitis according to the revised 2012 Atlanta guideline (9), who were admitted to the Ruijin Hospital, Shanghai Jiao Tong University School of Medicine, between January 2013 and December 2020, were enrolled in this study. Patient exclusion criteria included the following: (1) pregnancy; (2) history of pancreatitis or chronic pancreatitis; (3) hematological diseases or malignancy; (4) platelet count test <2 times within 3 days from admission; and (5) incomplete data. All enrolled patients were followed up until discharge or death.

### Data collection

The clinical variables are extracted from the electronic database for each patient. Baseline demographic information includes age, gender, body mass index, comorbidities (hypertension, diabetes, cerebrovascular diseases, cardiovascular diseases, chronic kidney diseases, and chronic obstructive pulmonary diseases), etiologies (biliary, hypertriglyceridemia, alcoholic, or others), and time interval from onset to admission. Laboratory indicators including blood amylase, white blood cell count, hemoglobin, fibrinogen, D-dimers, lactate, C-reactive protein, procalcitonin, alanine aminotransferase, alkaline phosphatase, pre-albumin, albumin, total bilirubin, creatinine, and Acute Physiology and Chronic Health Evaluation II scores (APACHE II) are collected within 24 h after admission. Platelet counts are collected within the first 3 days after admission. Thrombocytopenia is defined as a minimal platelet count of <100 × 10^9^/L within the first 3 days (10). Severities are classified as mild acute pancreatitis (MAP), moderately severe acute pancreatitis (MSAP), and severe acute pancreatitis (SAP) according to the revised 2012 Atlanta guideline (9). Clinical outcomes include the incidence of intra-abdominal infection, surgical intervention (percutaneous drainage and open abdominal debridement), in-hospital mortality, and length of hospital stay.

### Data statistics

Categorical data will be described with frequency or ratio. Continuous variables will be described using the median and interquartile range (IQR). Categorical variables will be compared using the chi-square test or Fisher exact test. Continuous variables will be compared using the *t*-test for normally distributed variables or Wilcoxon rank-sum test for non-normally distributed variables. The association between platelet count and mortality is estimated by logistic regression models, and the following confounders are adjusted: age, sex, body mass index, comorbidities, and etiologies. In addition, creatinine represents a renal function, C-reactive protein represents inflammation, procalcitonin represents infection, lactate represents oxygen debt, D-dimers represent hemostasis during inflammation, and the APACHE II score represents baseline conditions. These indexes are considered prognostic factors in previous studies (11, 12). Therefore, we also consider these indexes included in the regression models. The smoothing spline is plotted by utilizing a generalized additive model. A two-piecewise linear regression model is applied to examine the threshold effect of the platelet count on intra-abdominal infection and surgical intervention. All statistical analyses are performed using R software (version 4.2.1). A two-sided significance level of less than 0.05 is defined as statistical significance.

## Results

### Patient characteristics and laboratory findings

**FIGURE 1:**
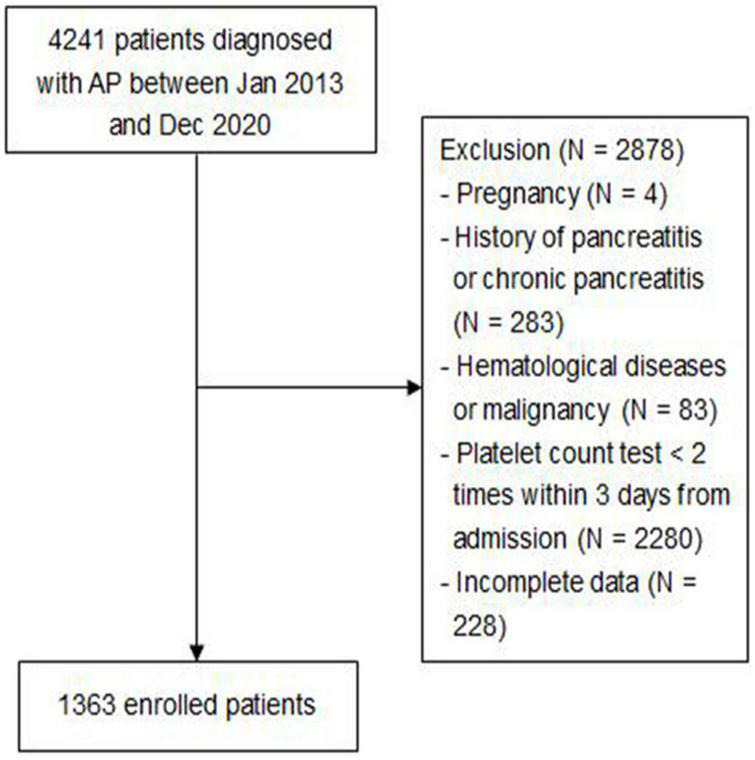
Screening flowchart.

A total of 4,241 patients diagnosed with AP between January 2013 and December 2020 were included, of which 2,878 patients were excluded based on exclusion criteria. Figure 1 shows the screening flowchart.

Among 1,363 enrolled patients, the median age is 48 years and 63.7% are male patients. The median time interval from onset to admission is 3 days (range 1–5 days). Patients with thrombocytopenia have a higher APACHE II score and are more likely to develop SAP. With a median of 21 days of hospital stay (range 11–36 days), 99 (7.3%) patients suffered intra-abdominal infection, 190 (13.9%) patients underwent surgical intervention, and 38 (2.8%) patients died in the hospital. Remarkable laboratory differences are also found in the levels of fibrinogen, D-dimers, lactate, procalcitonin, alanine aminotransferase, albumin, and creatinine (Table 1).

**TABLE 1:** Baseline characteristics of acute pancreatitis patients with thrombocytopenia and without thrombocytopenia.

	Total (*n* = 1363)	Thrombocytopenia (*n* = 178)	Non-thrombocytopenia (*n* = 1185)	*P-*value
Age, y	48.0 (37.0–62.0)	51.5 (40.0–64.0)	47.0 (36.0–62.0)	0.021
Gender (male %)	63.7 (868/1363)	68.0 (121/178)	63.0 (747/1185)	0.201
Body mass index, kg/m^2^	25.2 (23.0–27.4)	25.4 (23.0–27.1)	25.1 (23.0–27.4)	0.537
Time interval from onset to admission, days	3.0 (1.0–5.0)	3.0 (1.0–4.0)	3.0 (1.0–5.0)	0.241
**Comorbidities**				
Hypertension	31.9 (435/1363)	33.7 (60/178)	31.6 (375/1185)	0.582
Diabetes	25.1 (342/1363)	25.8 (46/178)	25.0 (296/1185)	0.804
Cerebrovascular diseases	2.6 (35/1363)	3.9 (7/178)	2.4 (28/1185)	0.217
Cardiovascular diseases	1.4 (19/1363)	1.7 (3/178)	1.4 (16/1185)	0.99
Chronic kidney diseases	1.1 (15/1363)	2.2 (4/178)	0.9 (11/1185)	0.235
COPD	1.5 (20/1363)	1.7 (3/178)	1.4 (17/1185)	1
**Etiologies**				
Biliary	48.3 (658/1363)	57.3 (102/178)	46.9 (556/1185)	0.01
Hypertriglyceridemia	31.6 (431/1363)	27.0 (48/178)	32.3 (383/1185)	0.152
Alcoholic	6.3 (86/1363)	3.9 (7/178)	6.7 (79/1185)	0.162
Others	13.8 (188/1363)	11.8 (21/178)	14.1 (167/1185)	0.408
**Laboratory indicators**				
Platelet count, ×109/L	164.0 (125.0–209.0)	78.0 (65.0–89.0)	176.0 (140.0–217.0)	<0.001
Amylase, U/L	421.0 (121.0–1126.0)	674.0 (186.0–1208.0)	386.0 (115.2–1063.0)	0.235
White blood cell, ×109/L	11.4 (8.3–15.1)	10.8 (7.9–14.2)	11.5 (8.4–15.2)	0.088
Hemoglobin, g/L	131.0 (115.0–146.0)	129.0 (110.0–149.1)	131.0 (116.0–145.0)	0.894
Fibrinogen, g/L	4.9 (3.4–6.2)	4.1 (3.0–5.7)	5.0 (3.6–6.2)	<0.001
D-dimers, mg/L	3.6 (1.6–6.4)	4.6 (2.5–8.8)	3.5 (1.5–6.1)	<0.001
Lactate, mmol/L	1.8 (1.5–2.4)	2.0 (1.5–3.0)	1.8 (1.5–2.3)	<0.001
C-reactive protein, mg/L	173.0 (83.5–243.0)	186.6 (106.4–264.5)	171.0 (83.0–240.0)	0.143
Procalcitonin, ng/mL	1.0 (0.4–2.5)	2.6 (0.7–6.1)	0.9 (0.4–2.2)	0.001
Alanine aminotransferase, IU/L	26.0 (16.0–62.5)	37.5 (18.2–83.5)	25.0 (15.0–57.0)	0.018
Alkaline phosphatase, IU/L	70.0 (54.0–99.0)	65.0 (51.0–94.5)	71.0 (55.0–100.0)	0.219
Pre-albumin, mg/L	137.0 (96.0–187.0)	133.0 (101.5–185.5)	137.0 (96.0–187.2)	0.709
Albumin, g/L	33.0 (29.0–37.0)	31.0(28.0–36.0)	34.0(30.0–38.0)	<0.001
Total bilirubin, μmol/L	21.5 (14.8–32.8)	27.6 (18.6–43.6)	21.0 (14.6–31.5)	0.052
Creatinine, μmol/L	66.0 (53.0–81.0)	72.0 (60.0–114.5)	65.0 (53.0–78.0)	<0.001
APACHEII score	7.0 (5.0–10.0)	8.5 (6.0–11.0)	7.0 (5.0–10.0)	<0.001
**Severities**				
MAP	39.6 (540/1363)	26.4 (47/178)	41.6 (493/1185)	<0.001
MSAP	47.0 (640/1363)	42.1 (75/178)	47.7 (565/1185)	0.167
SAP	13.4 (183/1363)	31.5 (56/178)	10.7 (127/1185)	<0.001
**Clinical outcomes**				
Intra-abdominal infection	7.3 (99/1363)	12.4 (22/178)	6.5 (77/1185)	0.005
Surgery intervention	13.9 (190/1363)	25.8 (46/178)	12.2 (144/1185)	<0.001
In-hospital mortality	2.8 (38/1363)	8.4 (15/178)	1.9 (23/1185)	<0.001
Hospital stay, days	21.0 (11.0–36.0)	27.0 (14.0–46.8)	21.0 (11.0–34.0)	<0.001

Data are presented as median and interquartile range. APACHE II score, Acute Physiology and Chronic Health Evaluation II score; COPD, chronic obstructive pulmonary disease; MAP, mild acute pancreatitis; MASP, moderately severe acute pancreatitis; SAP, severe acute pancreatitis.

### Association between platelet count and clinical outcomes

Logistic regression models are adjusted for several risk factors at admission, including age, sex, body mass index, comorbidities, etiologies, creatinine, C-reactive protein, procalcitonin, lactate, D-dimers, and APACHE II score. The results reveal after adjustment for potential confounders, the incidence of intra-abdominal infection is significantly lower in platelet count between 125 × 10^9^/L and 209 × 10^9^/L (4.1 and 3.5%) but significantly higher in platelet count lower than 125 × 10^9^/L (10.3%) or higher than 209 × 10^9^/L (11.1%). Similarly, the incidence of surgical intervention is significantly lower in platelet count between 125 × 10^9^/L and 209 × 10^9^/L (8.9 and 6.4%) and significantly higher in platelet count lower than 125 × 10^9^/L (22%) or higher than 209 × 10^9^/L (18.4%) (Table 2). However, after adjustment for potential confounders, platelet count has no significant effect on in-hospital mortality in each group.

**TABLE 2:** Association between platelet count and risk of intra-abdominal infection, surgical intervention, and in-hospital mortality estimated using logistic regression models.

	Quartile 1	Quartile 2	Quartile 3	Quartile 4	*p*-trend
Platelet count, ×109/L	<125	125–164	164–209	≥209	
No. of infection/total (%)	35/340 (10.3)	14/338 (4.1)	12/343 (3.5)	38/342 (11.1)	
Crude	1	0.38	0.32	1.09	0.772
	(Ref.)	0.2–0.71	0.16–0.62	0.67–1.77	
		0.003	0.001	0.73	
Model 1	1	0.37	0.31	1.07	0.871
	(Ref.)	0.2–0.71	0.16–0.61	0.65–1.75	
		0.003	0.001	0.795	
Model 2	1	0.36	0.29	1	0.945
	(Ref.)	0.19–0.69	0.15–0.58	0.6–1.66	
		0.002	<0.001	0.995	
Model 3	1	0.45	0.42	1.46	0.183
	(Ref.)	0.23–0.88	0.20–0.86	0.84–2.54	
		0.017	0.02	0.182	
No. of surgery/total (%)	75/340 (22.0)	30/338 (8.9)	22/343 (6.4)	63/342 (18.4)	
Crude	1	0.34	0.24	0.78	0.087
	(Ref.)	0.22–0.54	0.15–0.4	0.53–1.14	
		<0.001	<0.001	0.204	
Model 1	1	0.34	0.24	0.78	0.087
	(Ref.)	0.22–0.54	0.15–0.4	0.53–1.14	
		<0.001	<0.001	0.204	
Model 2	1	0.32	0.23	0.72	0.044
	(Ref.)	0.2–0.51	0.14–0.38	0.49–1.07	
		<0.001	<0.001	0.102	
Model 3	1	0.40	0.34	1.14	0.679
	(Ref.)	0.25–0.66	0.20–0.59	0.74–1.77	
		<0.001	<0.001	0.549	
No. of death/total (%)	22/340 (6.5)	7/338 (2.1)	5/343 (1.5)	4/342 (1.2)	
Crude	1	0.31	0.21	0.17	<0.001
	(Ref.)	0.13–0.73	0.08–0.57	0.06–0.5	
		0.007	0.002	0.001	
Model 1	1	0.3	0.23	0.19	<0.001
	(Ref.)	0.12–0.71	0.08–0.61	0.07–0.57	
		0.006	0.003	0.003	
Model 2	1	0.29	0.21	0.19	<0.001
	(Ref.)	0.12–0.7	0.08–0.57	0.06–0.56	
		0.006	0.002	0.003	
Model 3	1	0.82	0.87	0.67	0.57
	(Ref.)	0.26–2.56	0.26–2.95	0.18–2.45	
		0.73	0.819	0.543	

Data are presented as odds ratio (OR) and 95% confidence intervals. Model 1 adjusted for age, gender, and body mass index. Model 2 adjusted for Model 1, plus comorbidities (hypertension, diabetes, cerebrovascular diseases, cardiovascular diseases, chronic kidney diseases, and chronic obstructive pulmonary diseases) and etiologies (biliary, hypertriglyceridemia, alcoholic, or others). Model 3 adjusted for Model 2, plus creatinine, C-reactive protein, procalcitonin, lactate, D-dimers, and APACHE II score.

**FIGURE 2:**
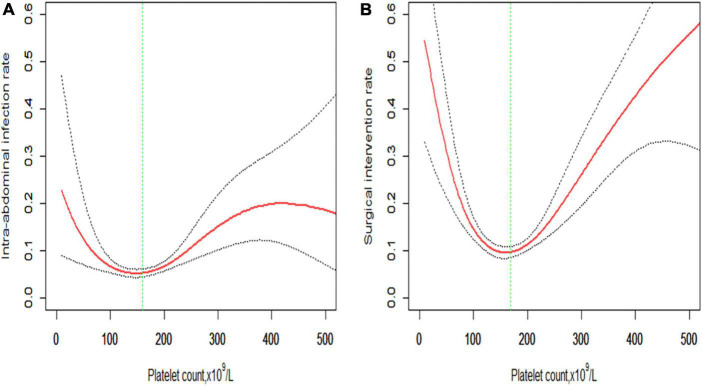
Non-linear relationship of **(A)** intra-abdominal infection rate and **(B)** surgical intervention rate with platelet count. The smoothing splines are generated utilizing a generalized additive model adjusted for age, gender, body mass index, comorbidities (hypertension, diabetes, cerebrovascular diseases, cardiovascular diseases, chronic kidney diseases, and chronic obstructive pulmonary diseases), etiologies (biliary, hypertriglyceridemia, alcoholic, or others), creatinine, C-reactive protein, procalcitonin, lactate, D-dimers, and APACHE II score. The red line indicates the estimated risk rate, the gray dot line indicates 95% confidence intervals, and the vertical green dot line indicates the inflection point.

The smoothing spline curve shows the non-linear relationship among the intra-abdominal infection rate (Figure 2A), surgical intervention rate (Figure 2B), and platelet count. In the threshold effect analysis, the risk of intra-abdominal infection decreases as the platelet count levels up to the inflection point (160 × 10^9^/L) (OR: 0.991, 95% CI: 0.984–0.998, *p* = 0.015). When the platelet count is 160 × 10^9^/L, the risk of intra-abdominal infection increases as the platelet count levels up (OR: 1.007, 95% CI: 1.004–1.010, *p* < 0.001). Similarly, the risk of surgical intervention decreases as the platelet count levels up to the inflection point (169 × 10^9^/L) (OR: 0.987, 95% CI: 0.982–0.992, *p* < 0.001). When the platelet count is 169 × 10^9^/L, the risk of surgical intervention increases as the platelet count levels up (OR: 1.010, 95% CI: 1.007–1.013, *p* < 0.001) (Table 3). The relationship (Supplementary Figure 1) between a hospital stay and platelet count is similar to the risk of intra-abdominal infection and surgical intervention. The inflection point of platelet count is 169 × 10^9^/L.

**TABLE 3:** Threshold effect analysis of the platelet count on intra-abdominal infection and surgical intervention.

Inflection point of platelet count, ×109/L	Increase per 1 × 109/L over the whole range of platelet count	*P*-value
**Intra-abdominal infection**		
≤160	0.991 (0.984–0.998)	0.015
>160	1.007 (1.004–1.010)	<0.001
**Surgical intervention**		
≤169	0.987 (0.982–0.992)	<0.001
>169	1.010 (1.007–1.013)	<0.001

Data are presented as odds ratio (OR) and 95% confidence intervals.

Further analysis of the changes in platelet count within the first 3 days after admission reveals that those changes have no obvious association with intra-abdominal infection, surgical intervention, and in-hospital mortality (Table 4).

**TABLE 4:** Association between the changes in platelet count within the first 3 days after admission and risk of intra-abdominal infection, surgical intervention, and in-hospital mortality estimated using logistic regression models.

	Quartile 1	Quartile 2	Quartile 3	Quartile 4	*p*-trend
Change in platelet count, ×109/L	<−22	(−22)–7.0	7.0–32.0	≥32.0	
No. of infection/total (%)	36/338 (10.6)	21/335 (6.3)	17/341 (5.0)	25/349 (7.2)	
Crude	1	0.56	0.44	0.65	0.065
	(Ref.)	0.32–0.98	0.24–0.8	0.38–1.1	
		0.043	0.007	0.11	
Model 1	1	0.57	0.44	0.64	0.062
	(Ref.)	0.33–1.01	0.24–0.81	0.37–1.1	
		0.052	0.008	0.106	
Model 2	1	0.55	0.47	0.66	0.101
	(Ref.)	0.31–0.98	0.25–0.86	0.38–1.15	
		0.042	0.015	0.141	
Model 3	1	0.70	0.58	0.75	0.301
	(Ref.)	0.38–1.28	0.30–1.11	0.42–1.37	
		0.245	0.099	0.357	
No. of Surgery/total (%)	64/338 (18.9)	43/335 (12.8)	33/341 (9.7)	50/349 (14.3)	
Crude	1	0.63	0.46	0.72	0.046
	(Ref.)	0.41–0.96	0.29–0.72	0.48–1.07	
		0.031	0.001	0.106	
Model 1	1	0.63	0.45	0.7	0.037
	(Ref.)	0.41–0.96	0.29–0.72	0.47–1.05	
		0.033	0.001	0.087	
Model 2	1	0.62	0.47	0.71	0.055
	(Ref.)	0.41–0.96	0.3–0.75	0.47–1.07	
		0.031	0.001	0.104	
Model 3	1	0.91	0.74	1.05	0.999
	(Ref.)	0.57–1.46	0.45–1.22	0.66–1.68	
		0.691	0.241	0.826	
No. of death/total (%)	19/338 (5.6)	8/335 (2.4)	6/341 (1.8)	5/349 (1.4)	
Crude	1	0.41	0.3	0.24	0.001
	(Ref.)	0.18–0.95	0.12–0.76	0.09–0.66	
		0.038	0.011	0.006	
Model 1	1	0.35	0.27	0.23	0.001
	(Ref.)	0.15–0.83	0.1–0.68	0.09–0.64	
		0.017	0.006	0.005	
Model 2	1	0.33	0.26	0.23	0.001
	(Ref.)	0.14–0.78	0.1–0.69	0.08–0.63	
		0.012	0.007	0.005	
Model 3	1	0.74	0.91	0.66	0.62
	(Ref.)	0.25–2.25	0.27–3.10	0.17–2.53	
		0.602	0.875	0.543	

Data are presented as odds ratio (OR) and 95% confidence intervals. Model 1 adjusted for age, gender, and body mass index. Model 2 adjusted for Model 1, plus comorbidities (hypertension, diabetes, cerebrovascular diseases, cardiovascular diseases, chronic kidney diseases, and chronic obstructive pulmonary diseases) and etiologies (biliary, hypertriglyceridemia, alcoholic, or others). Model 3 adjusted for Model 2, plus creatinine, C-reactive protein, procalcitonin, lactate, D-dimers, and APACHE II score.

## Discussion

Intra-abdominal infection is a detrimental factor of mortality in patients with acute pancreatitis (13). In the present study, we analyze the platelet count and changes in platelet count within 3 days after admission with the risk of intra-abdominal infection, surgical intervention, in-hospital mortality, and hospital stay. Logistic regression analysis and a generalized additive model are used and adjusted for several potential risk factors at admission. Our study highlights that, in patients with AP, (1) patients with platelet count on admission less than 125 × 10^9^/L or higher than 209 × 10^9^/L are more likely to suffer intra-abdominal infection, receive surgical intervention, and stay longer in the hospital; and (2) changes in platelet count within 3 days after admission have no significant association with intra-abdominal infection, surgical intervention, and in-hospital mortality.

Thrombocytopenia is recognized as an independent risk factor for prognosis. The estimated incidence in critically ill patients is 20–40% during hospitalization (7, 14). Thus, no study reported the incidence of thrombocytopenia in patients with AP. Among the enrolled 1,363 patients in our study, thrombocytopenia occurs in 178 (13%) patients within 3 days after admission. Patients with thrombocytopenia are more likely to deteriorate into SAP compared to patients with non-thrombocytopenia (31.5% vs. 10.7%). Intra-abdominal infection is one of the leading complications that can cause life-threatening organ dysfunction. Early identification of high-risk factors can save medical resources and potentially improve patient outcomes. In previous studies, procalcitonin is the most concerned predictor of intra-abdominal infection (15, 16). However, the small sample size (*n* < 200) in these studies limited the reliability. Moreover, procalcitonin is not a routine test in all hospitalized patients with AP. Recently, lactate is proven as a reliable predictor of intra-abdominal infection in Shu’s study (17). Even though the sample size is large enough (*n* = 503), the study design is not comprehensive because only patients with MSAP are included in this study. Prediction models are also constructed to screen the risk factors of intra-abdominal infection. In two multi-center retrospective studies, the platelet count is selected as a risk factor but not included in the final calculated models (18, 19). The reason may be that the linear models are used to select the risk factors. However, the relationship between platelet count and the risk of intra-abdominal infection is a non-linear pattern. In our study, the risk of intra-abdominal infection decreases as the platelet count levels up to 160 × 10^9^/L and then increases. The non-linear relationship is still significant after the adjustment of potential confounders, including age, gender, body mass index, comorbidities, etiologies, creatinine, C-reactive protein, procalcitonin, lactate, D-dimers, and APACHE II score. A two-piecewise linear regression model shows that the risk of intra-abdominal infection decreases by 0.9% per 1 × 10^9^/L increase in platelet when the platelet count is <160 × 10^9^/L, and increases by 0.7% per 1 × 10^9^/L increase in platelet when the platelet count is >160 × 10^9^/L. Interestingly, even the in-hospital mortality shows a declining trend in each quartile separated according to the platelet count (6.5, 2.1, 1.5, and 1.2%, respectively), the platelet count is not significantly associated with in-hospital mortality in patients with AP after adjustment for the confounders. The reason may be that the event (death) is relatively few in our study and, thus, the model is overfitted. In our study, we further analyze the dynamic changes in platelet count within the first 3 days after admission. No significant results are observed. In summary, the platelet count is a reliable and convenient factor for predicting intra-abdominal infection in patients with AP.

The mechanism of platelet count on intra-abdominal infection is poorly understood, but several explanations could be considered. The inflammatory process results in disturbances in pancreatic microcirculation, which has been recognized as an important factor in the development of acute pancreatitis (20). The ultra-structural changes in acute pancreatitis, including the infiltration of leukocytes, the accumulation of platelets intravascularly and extravascularly, and the formation of microthrombi, could be observed (21). However, the linear relationship between platelet count and some inflammatory indicators (C-reactive protein, white blood cell count, and procalcitonin) (Supplementary Figure 2) in this study could not provide a convincing explanation for the results. Another explanation is that the pancreatic vascular endothelial damage will activate the coagulation cascades that increase the consumption of platelet, which will exacerbate the pancreatic necrosis and eventually pancreas become infected. Interestingly, in this study, with the change in platelet count, the change trends of fibrinogen and D-dimers are consistent with the results (Supplementary Figure 3). It could be speculated that complex derangements of hemostasis occurred during acute pancreatitis. As a whole-body inflammatory disease, the damage of endothelial is not limited to the pancreatic tissues in severe pancreatitis. The multi-organ damage will further increase the activation of the platelet. The intestinal tract is the largest microorganism reservoir of the human body and could be easily damaged during systemic inflammation response. During the period of acute pancreatitis, the integrity of intestinal mucosa could be destroyed, which will also increase vascular permeability. Therefore, bacterial translocation could be easy. The decrease or increase in the platelet number may be the reflex of endothelial damage, which is observed in a previous study on sepsis (22). It is interesting that even though the risk of intra-abdominal infection is comparable between the low-level platelet count group (<125 × 10^9^/L) and high-level platelet count group (>209 × 10^9^/L) (10.3% vs. 11.1%) in our study, mortality is relatively low in high-level platelet count group (6.5% vs. 1.2%). The reason may be that a high platelet count reflects the better hematopoietic function of the body. Moreover, platelet possesses immune properties and can inhibit the spread of infection (23).

Our study is subjected to some limitations. First, as an observational study, it could not conclude that the associations between platelet count and clinical outcomes are causal. Second, the data were collected from one center, and the validation should be verified in other data sources. Third, the death event is relatively few in our study, and thus, the model may be overfitted. The association between platelet count and in-hospital mortality should be checked in a large number of patient studies.

## Conclusion

This retrospective cohort study among 1,363 patients with AP reveals that a platelet count of approximately 160 × 109/L on admission is associated with the lowest risk of intra-abdominal infection, surgical intervention, and shortest hospital stay in patients with acute pancreatitis. Moderate is the best.

## Data availability statement

The raw data supporting the conclusions of this article will be made available by the authors, without undue reservation.

## Ethics statement

The studies involving human participants were reviewed and approved by Institutional Ethics Board of Ruijin Hospital, Shanghai Jiao Tong University School of Medicine. The Ethics Committee waived the requirement of written informed consent for participation.

## Author contributions

JH and EM conceived the study. TN, YW, GM, WY, ZY, BZ, and YC helped in data collection. TN, BZ, HS, and LM helped in manuscript revision. WS, JH, TN, and YW were the co-first authors responsible for drafting and data analysis. EM was the responsible author. All authors contributed to the refinement of the study protocol. All authors contributed to the article and approved the submitted version.

## Supplementary material

Supplementary Figure 1 Non-linear relationship of hospital stay with platelet count. The smoothing splines are generated utilizing a generalized additive model adjusted for age, gender, body mass index, comorbidities (hypertension, diabetes, cerebrovascular diseases, cardiovascular diseases, chronic kidney diseases, and chronic obstructive pulmonary diseases), etiologies (biliary, hypertriglyceridemia, alcoholic, or others), creatinine, C-reactive protein, procalcitonin, lactate, D-dimers, and APACHE II score. The red line indicates the estimated hospital stay, the gray dot line indicates 95% confidence intervals, and the vertical green dot line indicates the inflection point.

Supplementary Figure 2 Smoothing spline presented the association between platelet count and **(A)** C-reactive protein; **(B)** white blood cell count; and **(C)** procalcitonin. Plots were generated by utilizing the generalized additive model.

Supplementary Figure 3 The smoothing spline presented the association between platelet count and **(A)** fibrinogen; **(B)** D-dimers. Plots were generated by utilizing the generalized additive model.
