# Supplementary figures and images for: Moderate level platelet count might be a good prognostic indicator for intra-abdominal infection in acute pancreatitis: A retrospective cohort study of 1,363 patients
Source: Front Med (Lausanne). 2023 Jan 9;9:1077076. doi: 10.3389/fmed.2022.1077076 (PMC9868935; doi:10.3389/fmed.2022.1077076)

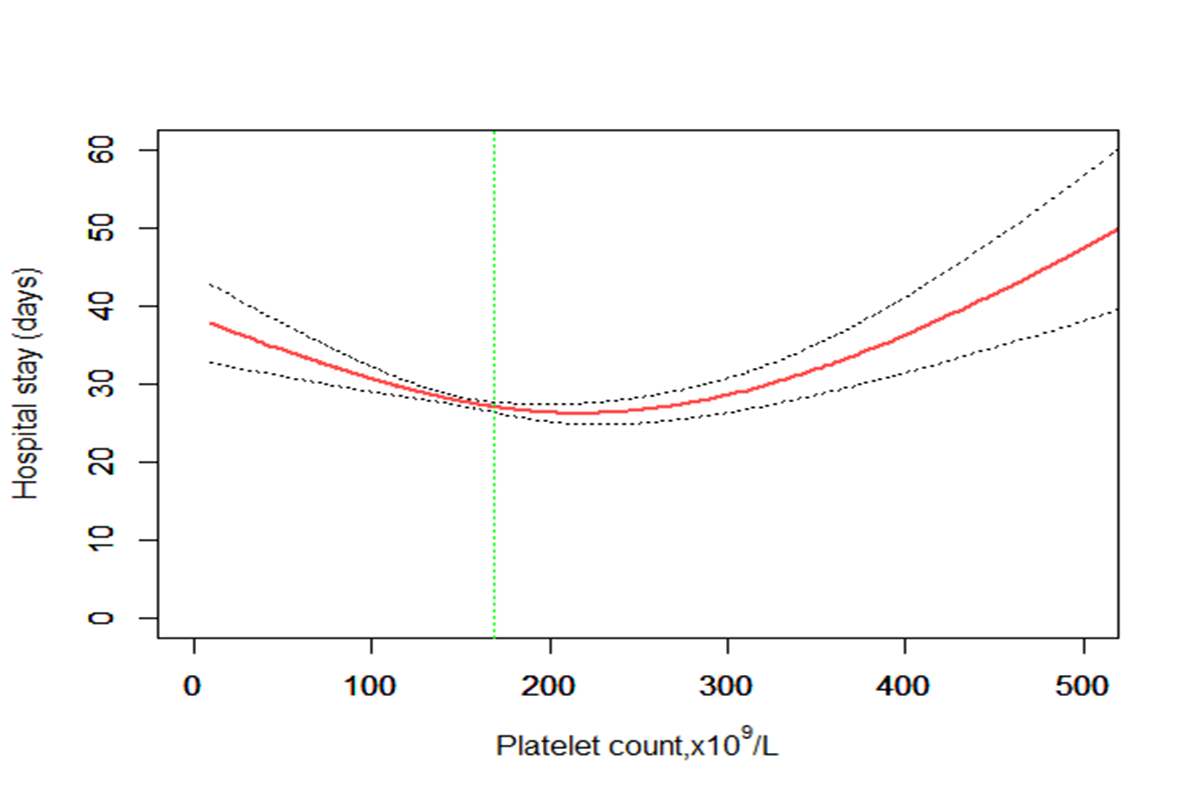

Supplement: Supplementary Figure 1 — Non-linear relationship of hospital stay with platelet count. The smoothing splines are generated utilizing a generalized additive model adjusted for age, gender, body mass index, comorbidities (hypertension, diabetes, cerebrovascular diseases, cardiovascular diseases, chronic kidney diseases, and chronic obstructive pulmonary diseases), etiologies (biliary, hypertriglyceridemia, alcoholic, or others), creatinine, C-reactive protein, procalcitonin, lactate, D-dimers, and APACHE II score. The red line indicates the estimated hospital stay, the gray dot line indicates 95% confidence intervals, and the vertical green dot line indicates the inflection point. [file Image_1.tif]

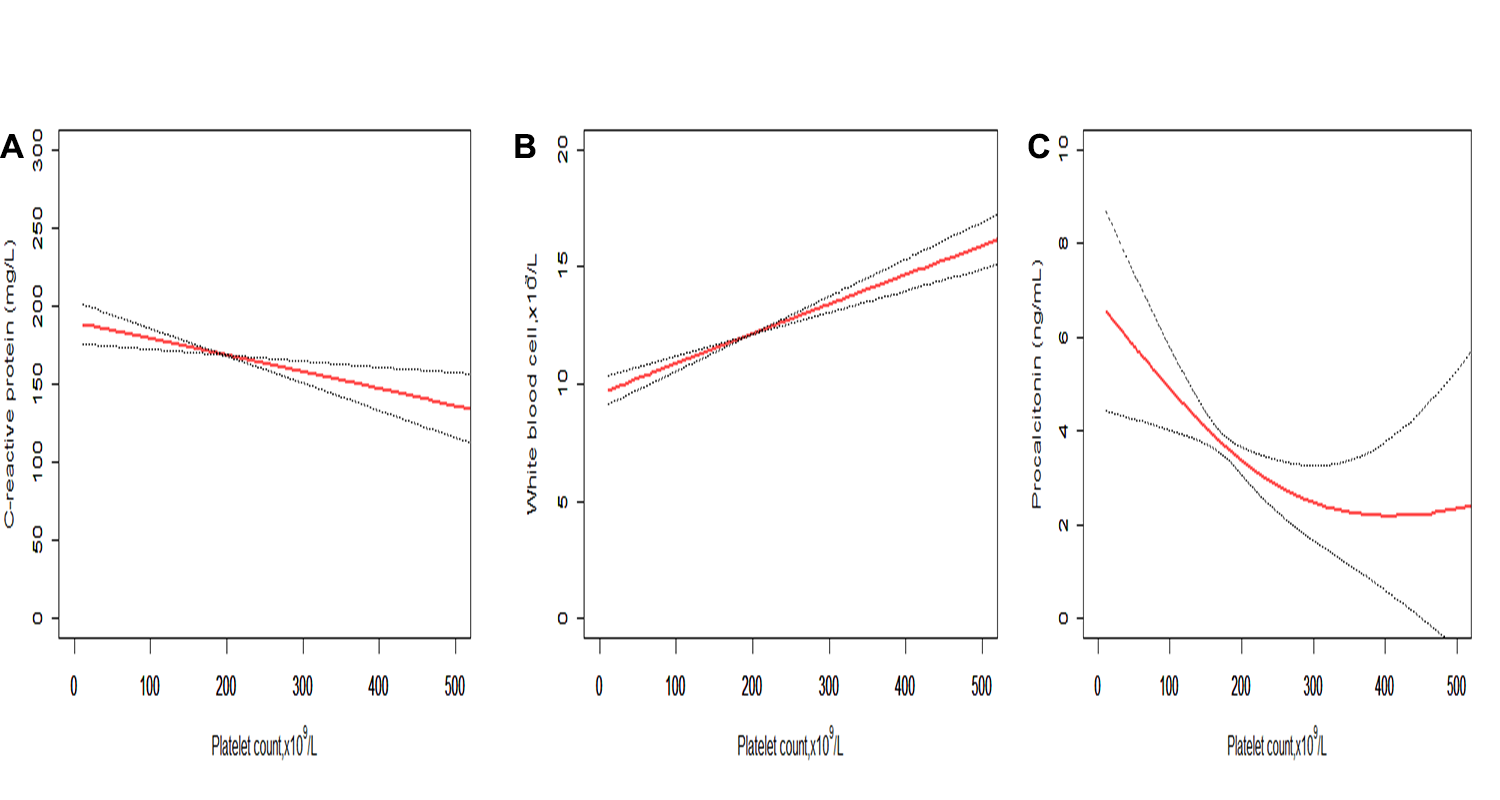

Supplement: Supplementary Figure 2 — Smoothing spline presented the association between platelet count and (A) C-reactive protein; (B) white blood cell count; and (C) procalcitonin. Plots were generated by utilizing the generalized additive model. [file Image_2.tif]

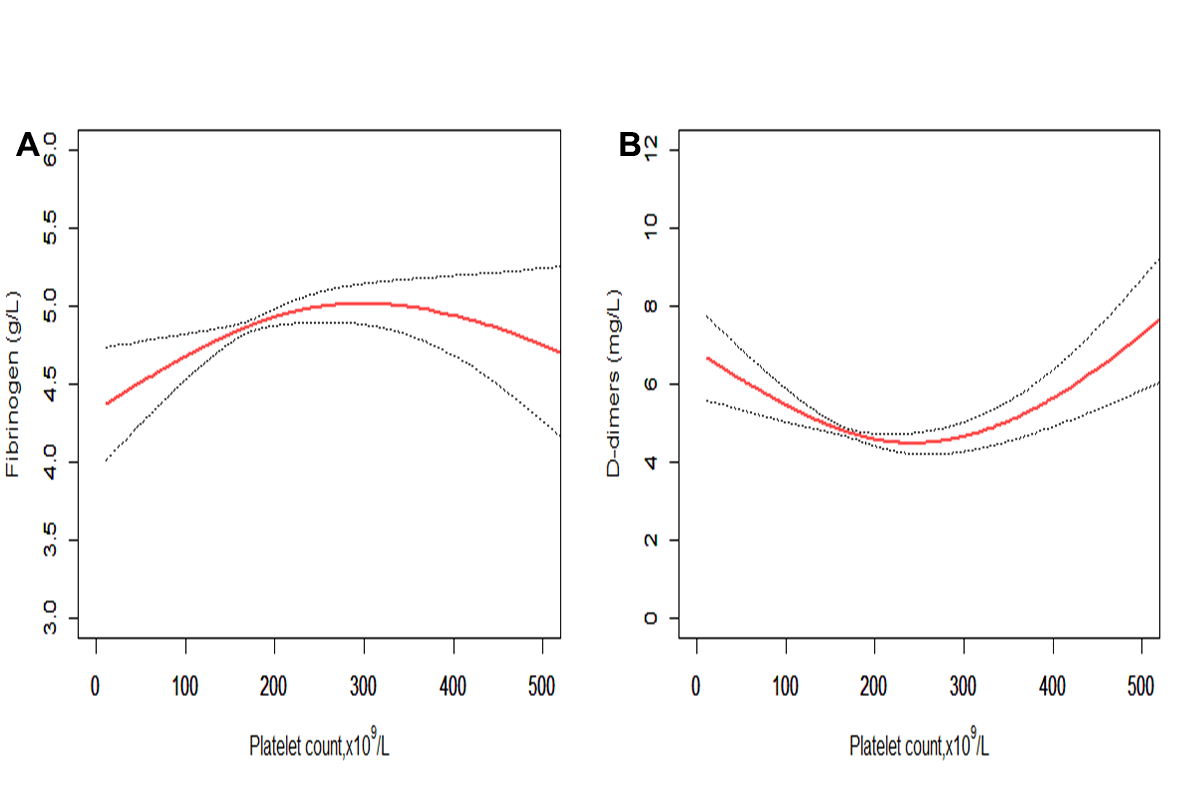

Supplement: Supplementary Figure 3 — The smoothing spline presented the association between platelet count and (A) fibrinogen; (B) D-dimers. Plots were generated by utilizing the generalized additive model. [file Image_3.tif]
